# Foreword

**DOI:** 10.1155/MBD.2000.iii

**Published:** 2000

**Authors:** Francisco González-Vílchez

**Affiliations:** Chemistry Faculty Inorganic Chemistry Department Bioinorganic Chemistry Section Campus “Reina Mercedes” Sevilla 41012 Spain


### Foreword

The late Prof. Marc Leng (Centre de Biophysique Moléculaire, CNRS, Orléans, France) died on 7th of May 2000 after a long cancerous process. His important contributions in different fields of Science as Biophysics, Biochemistry and Bioinorganic Chemistry constitute an impressive heritage to the scientific community and an indispensable and unique patrimony for actual and future generations of researchers. Although his international reputation was mainly due to the studies developed on DNA and its interaction with cisplatin and derivatives, including the corresponding mechanisms of action between these drugs and DNA, many other essential studies in the above mentioned fields, collected in more than 200 papers and three patents, contributed to the high scientific standing gained by Marc Leng.

Marc Leng was considered by his colleagues and disciples as a hard and constant worker, fond of nature and a scientist with a limitless curiosity. These so specific characteristics of Marc Leng reflected in daily life by the reading of many selected books and regular domestic gardening. He was also recognised by friends as an expert enologist, particularly fond of the growing and breeding of those wines coming from Bordeaux, his birthplace. This interest was manifested with joy recently with occasion of one of his stays in Spain where he visited Jerez, a place where the sherry wine grows and is particularly appreciated.

While the starting of Marc Leng in fundamental research happened during the accomplishment of his PhD studies at the CRM in Strasbourg, his interest in biological macromolecules and nucleic acids occurred after obtaining a postdoctoral fellowship at the laboratory of Dr. Garry Felsenfeld (NIH, Bethesda, USA), where he carried out a succesful research mainly on the interaction of DNA with polymers of lysine and arginine. His findings of the preferential interaction of polylysine with AT rich DNA regions were commented on in *Nature* [**223**, 1101, (1969)] and qualified as a remarkable discovery. The relationship and friendship stablished during that time between Dr. Fesenfeld and Marc Leng lasted until his last moments. Soon after returning to France he accepted the invitation of Charles Sadron, founder of the Centre de Biophysique Moléculaire in Orléans, to continue his research at this Centre, leading his own group. After his incorporation he showed for first time that the ordered form of polyuridilic acid results from the folding of the molecule on itself through base pairing. Again this finding was commented on in *Nature* (1970) [**227**, 22 (1970)].

In 1975, Marc Leng initiated a controversial project on the raising of anti-nucleic acids antibodies. This novel and very succesful research could be considered as the germ of the chemical carcinogenesis studies he commenced. Indeed, he used these antibodies in 1983 to develop a genetic immunodiagnostic technique that was patented in collaboration with the Institut Pasteur (Paris). After his excellent research on the existence of Z-DNA *in vivo* and its possible implication in cancer. He began his work on the mechanism of action of new promising platinum-based drugs, main focus of his attention for the next nineteen years. A varied and fruitful work was developed by Marc and his team on different aspects of anticancer compounds and their interactions with DNA. Among these, it is wort to mention the quantification of platinum adducts, the study of physico-chemical properties of platinated nucleic acids, the interactions between proteins and platinated DNA and the rearrangement of the transplatin 1,3-intrastrand cross-links into interstrand cross-links, this latter reaction used to modulate gene expression as part as an antisense strategy resulting in a recent patent.

Many merits adorned the scientific life of Marc. After been appointed director of research of the highest level, he got an important position at the direction of CNRS since 1999. He was co-director of postgraduate studies in Biologie-Biophysique Moléculaire et Cellulaire and lecturer at the University of Orléans-Tours. He directed and supervised 30 theses of national and foreign students and was the coordinator of the Biomed 2 european contract and member of the Cost Action D8 project "Metal in Medicine". He was an editorial board member of "Metal-Based Drugs" journal since 1994.

Colleagues, friends and scientists all over the world will never forget his memory. The death of this pioneer of the Biological Inorganic Chemistry is a irreparable loss for the scientific community as a whole.

Francisco González-Vílchez
Chemistry Faculty
Inorganic Chemistry Department
Bioinorganic Chemistry Section
Campus "Reina Mercedes"
41012 SEVILLA (Spain)

